# Plasma-Derived Neuronal Exosomal CircRNAs as Potential Biomarkers for Central Nervous System Infections

**DOI:** 10.1155/jimr/9363390

**Published:** 2025-02-25

**Authors:** Rui Zhang, Bihan Deng, Shuaibing Shi, Geng Lu, Jun Xia, Hongwei Liang, Fei Liu, Shuangshuang Gu, Jun Wang

**Affiliations:** ^1^Department of Emergency Medicine, Nanjing Drum Tower Hospital, Nanjing Drum Tower Hospital Clinical College of Nanjing University of Chinese Medicine, Nanjing, China; ^2^Department of Emergency, Nanjing Drum Tower Hospital, Medical School of Nanjing University, Nanjing, China; ^3^Nanjing Drum Tower Hospital Clinical College of Xuzhou Medical University, Nanjing, China; ^4^Department of Emergency, Nanjing Drum Tower Hospital, School of Life Science and Technology, China Pharmaceutical University, Nanjing, China

## Abstract

Infections of central nervous system (ICNSs) are inflammatory diseases caused by infectious agents that can infiltrate the brain and spinal cord through various routes, including the bloodstream, peripheral nerves, or cranial nerves. Exosomes are found in plasma and have the capacity to cross the blood–brain barrier (BBB). Exosome constituents, including lipids, proteins, DNA, and RNA, change significantly over time and are correlated with the course of disease. Circular RNA (circRNA) has become a potential biomarker for various diseases, such as ICNSs. This study explores the diagnostic potential of circRNAs derived from brain-derived exosomes in ICNSs. Our research shows that the brain-derived exosomes from patients with CNS illnesses have different patterns of circRNA expression than those from healthy controls. Plasma samples from patients with bacterial ICNSs show significantly elevated levels of hsa_circ__0020840 and hsa_circ_0116108. In contrast, higher expression levels of hsa_circ_0056947 and hsa_circ_0021531 are observed in plasma samples from individuals with viral ICNSs compared to healthy subjects. These observations suggest their potential utility as sensitive and specific biomarkers for these diseases. Moreover, the capacity of circRNAs to be encapsulated within exosomes and released into circulation offers a noninvasive approach for diagnosing ICNSs. These findings highlight the promise of utilizing brain-derived exosomal circRNAs as novel diagnostic markers for ICNSs, which may have implications for improving patient outcomes and disease management.

## 1. Introduction

Central nervous system infections (ICNSs) are a major risk to human health, frequently causing serious neurological problems and possible death if not properly identified and treated. Viruses [1] and bacteria [2] are commonly acknowledged as the main causal agents of CNS illnesses. People who are suspected of having an ICNS usually have severe symptoms, such as changes in consciousness, neurological problems, and unstable blood pressure. Neurological Imaging methods, such as computerized tomography (CT) and MR imaging (MRI), along with certain cerebrospinal fluid (CSF) analyses, are essential for detecting abnormalities in the CNS [1]. MRI demonstrates greater sensitivity and specificity [3] compared to CT for early detection of encephalitis; however, it may not significantly contribute to etiological differentiation initially when findings appear normal. Additionally, resource shortages and suboptimal medical facilities in certain regions can compromise the advantages of MRI. Therefore, lumbar puncture (LP) is commonly performed on most patients suspected of meningitis unless there are clear contraindications to analyze CSF. However, LP carries contraindications and adverse effects such as headaches or abnormal sensations. ICNS symptoms may not be apparent in the early stages, posing a diagnostic challenge due to various potential conditions ranging from bacterial meningitis and septic encephalopathy to migraines or systemic viral infections. Prompt diagnostic evaluation, identification of elements unique to the cause, and timely initiation of focused treatment are essential for enhancing outcomes. However, the clinical criteria and further examinations frequently do not possess the essential sensitivity and specificity to effectively differentiate between these many causes. Delayed or needless administration of antibiotics or antiviral medications may result from the challenge of promptly diagnosing the condition. Identifying high-risk categories linked to negative outcomes is essential for maximizing results in this group of patients.

Plasma/serum exosomes, extracellular vesicles (EVs) released by cells, encompass a diverse range of intracellular macromolecules, including lipids, proteins, and nucleic acids [4]. Recent investigations have uncovered the vital function of plasma exosomes in facilitating communication and transmission of information between cells [5–7]. Exosomes derived from diseased tissues have emerged as a rich source of biomarkers [8]. For instance, immunocapture of circulating EVs presumed to be neuronally derived using anti-L1CAM (L1EVs) has been employed as a proxy biomarker for brain pathology in various neurological disorders [9]. Within these exosomes, circular RNAs (circRNAs), a class of regulatory noncoding RNAs, have garnered significant attention due to their potential as disease biomarkers owing to their remarkable stability, specific expression patterns, and evolutionary conservation [10–12]. Thus, circRNAs hold promise as diagnostic tools for diseases and can aid in treatment selection and prognosis.

We hypothesize that circRNA present in exosomes derived from brain tissue in the bloodstream could potentially serve as a biomarker for ICNSs. This study involved an initial investigation of the expression profiles of circRNA in exosomes obtained from brain tissue in serum. This investigation was conducted using high-throughput sequencing. Subsequently, the dysregulated circRNAs were verified by quantitative RT-PCR (qRT-PCR) analysis. Our findings indicate that hsa_circ__0020840, hsa_circ_0116108, hsa_circ_0021531, and hsa_circ_0056947 present in exosomes derived from brain tissue in the blood may serve as sensitive biomarkers for ICNS.

## 2. Materials and Methods

### 2.1. Study Procedure

**Figure 1:**
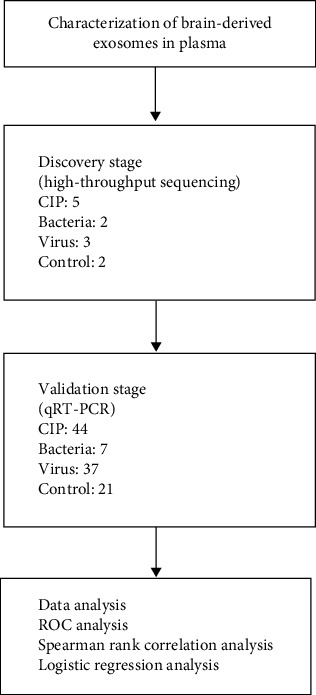
The flowchart depicting the methodology employed in this study.

We performed a three-stage study to confirm the candidate biomarkers for ICNS (Figure 1). First, the brain-derived plasma exosomes were isolated and identified by western blotting, transmission electron microscopy (TEM), and nanoparticle tracking analysis (NTA). Then, the RNAs in the brain-derived plasma exosomes of five patients with ICNS (CIP), including three viruses or two bacteria infection patients and two patients exhibiting symptoms resembling ICNSs (control), were performed in high-throughput sequencing. Finally, the selected candidate biomarkers through high-throughput sequencing were validated by qRT-PCR in 44 patients with ICNSs caused by viruses (*n* = 38) or bacteria (*n* = 6) (patients), and 21 patients exhibiting symptoms resembling ICNSs (control). The ROC analysis, Spearman rank correlation analysis, and logistic regression analysis were further performed to analyze the results of qRT-PCR.

### 2.2. Patients and Healthy Controls

Serum samples were collected upon the initial admission to Nanjing Drum Tower Hospital. Subsequently, these samples were categorized into groups based on their final clinical diagnosis: 44 patients with ICNSs caused by viruses (*n* = 38) or bacteria (*n* = 6) (patients), and 21 patients exhibiting symptoms resembling ICNSs (control).  Table 1 presents the demographic and clinical characteristics of patients diagnosed with viral or bacterial ICNSs, as well as those with comparable symptoms. The research protocol received approval from the Institutional Review Board at Nanjing Drum Tower Hospital and was conducted in accordance with the principles of the Helsinki Declaration.

### 2.3. Plasma Collection

Blood was obtained through venipuncture using a BD Vacutainer push-button blood-collection kit and left to clot in silicone-coated serum-collection tubes for 20 min. Afterward, the serum was acquired using centrifugation with a force of 1500 × *g* for a duration of 20 min at a temperature of 4°C. Further centrifugation steps were performed at 3000 × *g* and 10,000 × *g* for 20 min each, both at 4°C to eliminate cellular debris. The serum obtained was immediately divided into Eppendorf Safe-Lock Tubes (1.5 mL) and frozen at −80°C.

### 2.4. Isolation of Exosomes Derived From Brain Tissues

To isolate neuronal, astrocytic, and microglial exosomes, 2 µg each of anti-L1CAM (L1 cell-adhesion molecule), anti-GLAST (glutamate aspartate transporter), and anti-TMEM119 (transmembrane protein 119) antibodies were individually coated onto 1 mg of M-270 epoxy Dynabeads (Cat#14302D, Life Technologies, USA) using a Dynabeads Antibody Coupling Kit (Cat#14311D, Life Technologies, USA). The coating was performed overnight at 37°C with gentle rotation, following the manufacturer's instructions. The Dynabeads with anti-L1CAM, anti-GLAST, and anti-TMEM119 antibodies were then combined in equal proportions to create a mixed bead solution for isolating exosomes derived from brain tissue.

Frozen serum samples were thawed on ice and then mixed gently with 500 µL of serum and 100 µL of Total Exosome Isolation Reagent (Cat# 4478360, Invitrogen, USA). This mixture was incubated at 4°C for 30 min. After incubation, the exosomes were separated by centrifugation at 10,000 × *g* for 10 min at 4°C, resulting in the formation of a pellet at the bottom of the tube. After carefully aspirating and discarding the supernatant, the exosomes were completely resuspended in 50 µL of PBS. The resuspended exosomes were mixed gently with the combined Dynabeads and incubated at 4°C for 4 h with gentle rotation. In the final step, exosomes bound to beads were rinsed two times with 1 mL of PBS supplemented with 0.1% (*w*/*v*) BSA before being transferred to fresh tubes for collection.

### 2.5. Exosome Isolation and Characterization

Exosomes extracted from immunomagnetic beads and from the exosome extraction kit were characterized using NTA (NanoSight NS300), TEM, and western blot.

### 2.6. RNA Isolation

RNA isolation from exosomes extracted with mixed Dynabeads was conducted using the mirVana PARIS Kit (Ambion, Thermo Scientific, Shanghai, China). To standardize the qRT-PCR results, 5′-UCACCGGGUGUAAAUCAGCUUG-3′, a synthetic microRNA from *Caenorhabditis elegans* (RiboBio, Guangzhou, China), was included in the denatured exosomes. The isolated total RNA was resuspended in 20 µL of RNase-free water. The quality and concentration of the RNA were evaluated using a NanoDrop 2000 spectrophotometer (Thermo Scientific) [13].

For cDNA synthesis from the exosome-derived RNA, HiScript III All-in-one RT SuperMix (Cat# R333, Vazyme, Nanjing, JS, China) was utilized. qRT-PCR reactions were conducted using the Roche LightCycler 96 instrument with AceQ Universal SYBR qPCR Master Mix (Cat# Q511, Vazyme, Nanjing, JS, China) according to the manufacturer's protocols. The reverse transcription reactions consisted of a 10 µL solution containing 4 µL of extracted RNA, 2 µL of 5× All-in-one qRT SuperMix, 1 µL of enzyme mix, and 3 µL of RNase-free ddH_2_O. The reactions were incubated at 50°C for 15 min, followed by a heat inactivation step at 85°C for 1 min [13].

The PCR reaction was set up with a total volume of 10 µL, comprising 1 µL of cDNA, 5 µL of 2× AceQ Universal SYBR qPCR Master Mix, 0.5 µL of a primer mixture (10 µM), and 3.5 µL of ddH_2_O. The amplification protocol commenced with an initial denaturation at 95°C for 5 min, then progressed through 40 cycles consisting of denaturation for 10 s at 95°C and annealing/extension at 60°C for 30 s. Each reaction, including controls without templates, was performed in triplicate to ensure reproducibility. The average CT value was calculated from the triplicate reactions for each sample. The primers used in this study were synthesized by Tsingke Biotechnology Co., Ltd. (Nanjing, China) [13].

### 2.7. Statistical Analyses

Data analysis was performed using SPSS version 18.0 (SPSS Inc., Chicago, IL, USA). Graphs were generated with GraphPad Prism version 6.0 from GraphPad Software, San Diego, CA, USA. The Mann–Whitney *U* test was employed to assess differences between groups, with a *p*-value of less than 0.05 considered statistically significant [13, 14].

## 3. Results

### 3.1. Identification of Brain-Derived Plasma Exosomes

**Figure 2:**
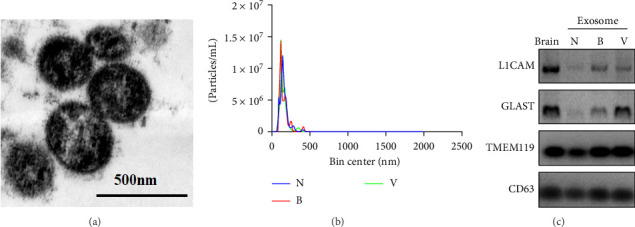
The identification of brain-derived plasma exosomes. (A) Exosomes under electron microscope. (B) Distribution of exosome size. (C) The identification of exosomal protein markers.

The plasma exosomes isolated by Dynabeads coated with anti-L1CAM, anti-GLAST, and anti-TMEM119 were characterized using the techniques of western blotting and NTA, which are well-established and frequently employed methods in the scientific community (Pegtel and Gould, 2019). TEM, as well as NTA, revealed that the exosomes exhibited an average diameter of ~100 nm, consistent with previous findings (Figure 2A,B). Additionally, western blot analysis validated the identification of CD63 as a marker for exosomal proteins, demonstrated in Figure 2C. Collectively, these results from western blotting, TEM, and NTA provide strong evidence validating the successful isolation of exosomes from serum. We also confirmed the detection of neuron-specific proteins, including L1CAM, GLAST, and TMEM119, within the isolated exosomes. This compelling evidence supports our claim that these isolated exosomes originate from neuronal cells.

### 3.2. Filtering for Differentially Expressed CircRNAs

**Figure 3:**
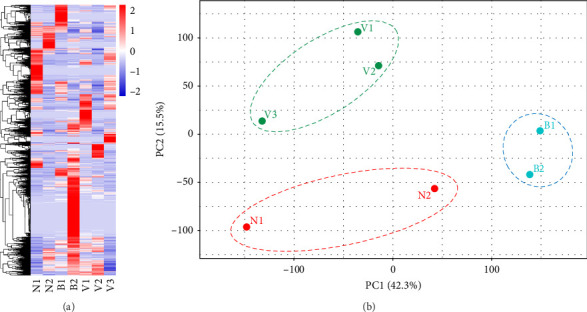
Expression profiling and cluster analysis of circRNAs in patients with non-ICNS patients, bacterial ICNSs, and viral ICNSs. (A) The high-throughput sequencing and cluster analysis of three groups. (B) PCA plot of non-ICNS patients, bacterial ICNSs, and viral ICNSs. CircRNAs, circular RNAs; ICNS, infections of central nervous system.

High-throughput sequencing was employed to analyze samples from two healthy controls and five disease groups, including two bacterial ICNSs and three viral ICNSs. A total of 52,362 circRNAs were identified in the samples from healthy human controls, viral ICNSs, and bacterial ICNSs. Among these circRNAs, there were 223 differentially expressed circRNAs in the viral central infection (54 upregulated and 169 downregulated), as well as 773 differentially expressed circRNAs in the bacterial central infection group (698 upregulated and 75 downregulated), when compared to healthy controls using a fold change ≥2 and *p*-value <0.05 (Table S1). Hierarchical cluster analysis was performed on all differentially expressed circRNAs, and their gene expression patterns were visualized through a heat map (Figure 3A). The PCA plot clearly demonstrated distinct clustering patterns among patients with virus infection, bacterial infection, and control subjects (Figure 3B). Each group formed a well-defined cluster without any sample overlap, indicating significant differences in gene expression patterns among these groups.

### 3.3. Verification of Differentially Expressed CircRNAs

**Figure 4:**
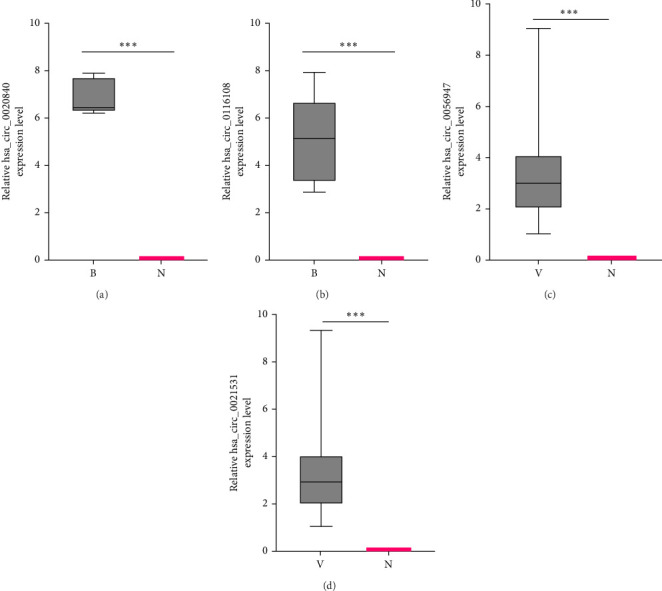
The level of target cyclic RNA expression was verified by RT-qPCR. The quantities of hsa_circ_0020840 (A) and hsa_circ_0116108 (B) in bacterial central infections were compared to healthy controls (N). Similarly, the amounts of hsa_circ_0056947 (C) and hsa_scirc_0021531 (D) in viral neutrophilic infections were assessed against healthy controls. *⁣*^*∗∗∗*^*p* < 0.001.

PCR validation was conducted on four circRNAs, chosen from a total of 773 and 223 cyclic RNAs that were found to be significantly different in bacterial and viral central infections, respectively, in this work.  Figure 4 shows a significant increase in the expression levels of hsa_circ_0020840 and hsa_circ_0116108 in plasma exosomes of individuals with bacterial central infections when compared to healthy controls. Similarly, hsa_circ_0056947 and hsa_circ_0021531 exhibited notable elevations in patients with viral central infections in contrast to healthy subjects.

### 3.4. Gene Ontology (GO) and Kyoto Encyclopedia of Genes and Genomes (KEGG) Analysis of Target Genes

In our study of the differential gene expression in brain-derived exosomes from the plasma of patients with bacterial and viral ICNSs, we conducted GO and KEGG pathway analyses that revealed significant enrichment of multiple biological processes and signaling pathways. Notably, dendritic transport and insulin metabolic processes were prominently regulated in both patient groups, suggesting their crucial roles in the immune response within the CNS. Additionally, the regulation of hepatocyte apoptotic processes and the positive regulation of the Hippo signaling pathway were significantly enriched, indicating that these mechanisms may be closely related to the regulation of cell survival and proliferation. We also found that the catabolism of cyclic nucleotides plays an important role in cellular signaling, potentially influencing neuronal function and survival. At the molecular level, the enrichment of N6-methyladenosine RNA binding and the phospholipid-translocating ATPase complex further underlines the significant roles these molecules may play in the biosynthesis and function of exosomes.

## 4. Discussion

**Figure 5:**
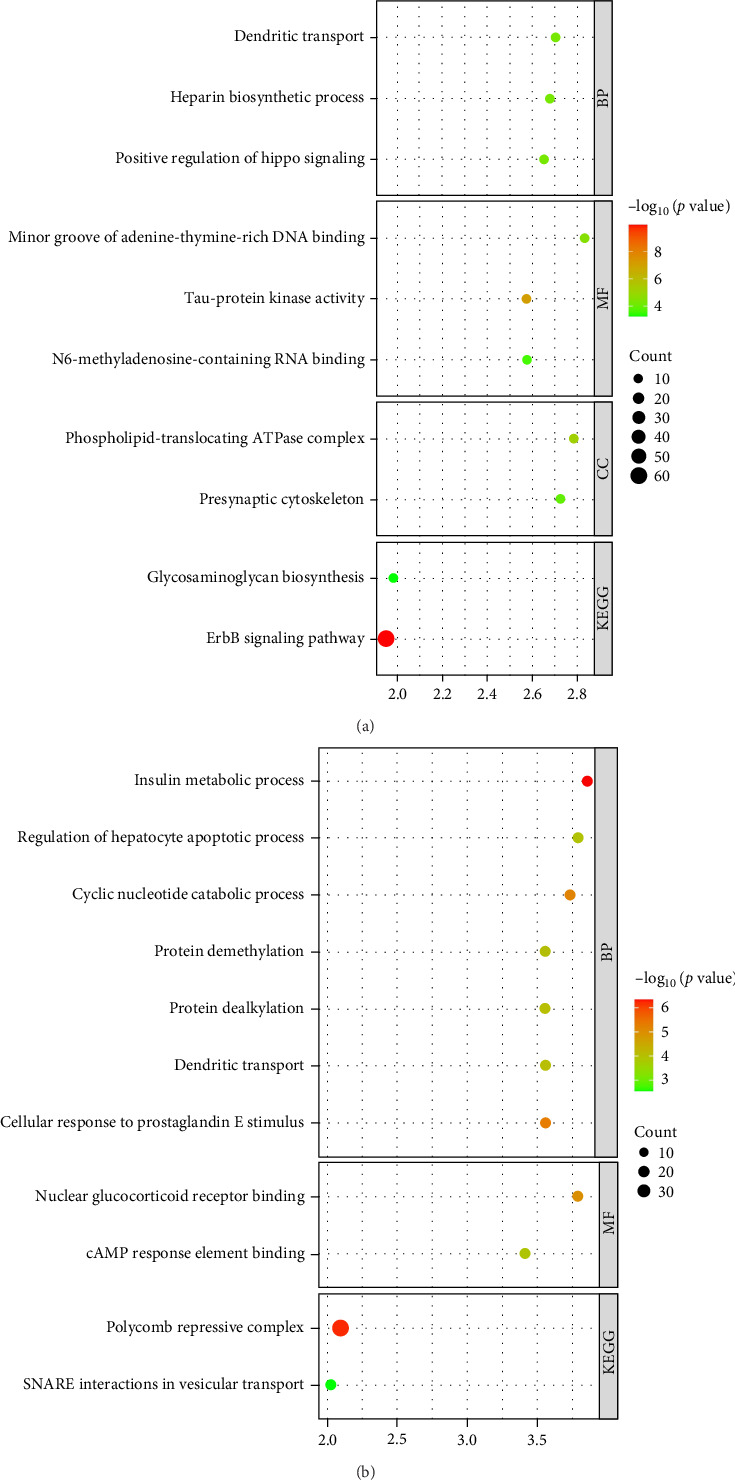
GO and KEGG analysis of target genes. PANTHER analysis performed a GO and KEGG annotation of the hsa_circ_0020840 and hsa_circ_0116108 in bacterial central infections (A), and hsa_circ_005694 and hsa_circ_0021531 in viral central infections (B). GO, Gene Ontology; KEGG, Kyoto Encyclopedia of Genes and Genomes.

CircRNA, a highly conserved noncoding RNA, has emerged as a robust marker for disease diagnosis [15]. Hu et al. [16] demonstrated that hsa_circ_0036877 has promise as an early diagnostic marker for pre-eclampsia. Additionally, exosomal circRNA has shown promise in diagnosing gestational diabetes, pancreatic cancer, and NAFLD [17–19]. In this study, we report that plasma brain-derived exosomal cyclic RNA can serve as a diagnostic biomarker for central infections. We isolated brain-originated exosomes and identified 52,362 differentially expressed circRNAs using high-throughput sequencing. Notably, two circRNAs, hsa_circ_0020840 and hsa_circ_0116108, showed a marked increase in expression during bacterial central infections, while hsa_circ_005694 and hsa_circ_0021531 demonstrated significant elevation in viral central infections. Furthermore, circRNAs have been shown to play significant roles in the initiation and progression of ICNS, [20, 21] such as hsa_circ_0116108 (also named circPKNOX1) affected the progression of intervertebral disc disease by regulating the expression of KIAA0355 via miR-370-3p [22]. GO and KEGG pathway analyses were conducted to elucidate the functional roles of dysregulated circRNAs in plasma exosomes. Utilizing the PANTHER database for this investigation, we found that the hsa_circ_0020840 and hsa_circ_0116108 were involved in the positive regulation of hippo signaling, glycosaminoglycan biosynthesis, etc., while the hsa_circ_005694 and hsa_circ_0021531 were involved in cAMP response element binding, SNARE interactions in vesicular transport, etc. These findings suggest that the differentially expressed circRNAs in exosomes may play a pivotal role in ICNS (Figure 5).

This research offers important perspectives on the potential role of brain-derived exosomal circRNAs as diagnostic markers for ICNSs. Our results demonstrate that circRNAs present in brain-derived exosomes exhibit distinct expression patterns in patients with ICNSs compared to healthy controls, indicating their potential role as biomarkers for these conditions. One of the key findings of our study is the identification of specific circRNA signatures that are differentially expressed in brain-derived exosomes from patients with ICNSs. This highlights the potential utility of circRNAs as highly sensitive and specific biomarkers for the early detection and diagnosis of ICNSs. The observed variations in circRNAs expression within brain-derived exosomes may provide valuable insights into the underlying pathological mechanisms associated with ICNSs. Furthermore, the ability of circRNAs to be encapsulated within exosomes and subsequently released into the systemic circulation makes them attractive candidates for noninvasive diagnostic testing [23]. The detection of circRNAs in circulating exosomes offers a minimally invasive approach for diagnosing ICNSs, potentially overcoming the limitations associated with current diagnostic methodologies that require invasive procedures such as CSF analysis.

Exosomes have emerged as a promising avenue for clinical diagnosis and biomarker discovery, offering a number of potential advantages. First, the composition of exosomes, which includes lipids, proteins, and nucleic acids, is known to undergo modifications during the progression of disease [24]. This offers the potential for insights to be gained into the disease state. Second, exosomes can be extracted from easily accessible biological fluids such as blood, urine, and saliva without the need for invasive procedures, which is particularly advantageous for early diagnosis, especially in conditions affecting the CNS [25, 26]. Third, their double-layered membrane provides protection for potential biomarkers from degradation. Furthermore, exosomes demonstrate high stability, rendering them suitable for clinical utilization, as samples can be stored for extended periods prior to analysis. Fourth, exosomes are capable of carrying specific markers that are indicative of their cellular origins, thereby facilitating the traceability of biomarkers. Lastly, their capacity to traverse the blood–brain barrier (BBB) enables the acquisition of data pertaining to neural cells that would otherwise be challenging to obtain through invasive methods. The blood serves as a vital medium that interacts with all organs and harbors extensive information about the organism. Neurons and glial cells secrete EVs, which play significant roles in both physiological and pathological processes associated with brain diseases. These EVs enable bidirectional communication between the CNS and peripheral systems due to their unique capacity to traverse the BBB [27]. Consequently, EVs derived from neurons and glial cells in the bloodstream present intriguing biomarker opportunities for the minimally invasive diagnosis of CNS disorders [28]. In particular, plasma brain-derived exosomal circRNAs demonstrate considerable potential for incorporation into clinical practices pertaining to ICNSs [29]. They serve as noninvasive biomarkers for diagnosis, prognosis, and treatment response. Their inherent stability in bodily fluids facilitates real-time monitoring of disease progression and immune response, thereby enabling clinicians to devise bespoke treatment strategies for individual patients. For instance, specific circRNA profiles could be employed to differentiate between viral and bacterial infections, thereby guiding the selection of appropriate antibiotics or antiviral therapies. Furthermore, monitoring circRNA level fluctuations during treatment could inform necessary adjustments to therapeutic regimens, thereby improving patient management and outcomes by mitigating complications arising from inappropriate treatments. Ultimately, the incorporation of plasma exosomal circRNAs into clinical practices could foster more informed, timely, and personalized approaches to managing ICNSs.

The measurement of plasma brain-derived exosomal hsa_circ_0020840 and hsa_circ_0116108 may potentially serve as biomarkers for bacterial ICNS, while hsa_circ_005694 along with hsa_circ_0021531 could indicate viral ICNSs. However, the current study is constrained by its small sample size. It is, therefore, imperative that larger cohort studies be conducted to validate the utility of these biomarkers in assessing risk and monitoring patients with ICNSs. Furthermore, it should be noted that our research has not investigated the functional roles of plasma brain-derived exosomal circRNAs in the context of ICNSs.

Although the plasma brain-derived exosomal circRNAs showed strong potential as an ideal diagnostic biomarker for ICNS, the use of exosomal circRNAs as diagnostic biomarkers still presents several potential challenges and limitations. First, although exosomal circRNAs are generally more stable than linear RNAs due to their circular structure, environmental factors such as temperature fluctuations and prolonged storage conditions can still lead to degradation. Ensuring the stability of these biomarkers during collection, processing, and storage is critical for accurate analysis. Second, variability in exosomal circRNA extraction and quantification methods can lead to inconsistent results. Standardization of protocols for isolating exosomes and measuring circRNA levels is essential to ensure reproducibility across different laboratories and studies. Third, the quality of biological fluids used for isolation (e.g., blood, urine, and saliva) can affect the yield and purity of exosomal circRNAs. Contaminants from other cellular components or EVs may interfere with the detection and quantification of circRNAs, potentially leading to false positives or negatives. Fourth, individual differences in physiology, disease state, and genetic background can influence the expression levels of circRNAs in exosomes. Factors such as age, sex, and comorbidities may introduce variability that complicates the interpretation of circRNA levels as diagnostic indicators. Fifth, while certain circRNAs may be associated with specific diseases, their expression could also be influenced by other physiological or pathological conditions. This lack of specificity could limit their utility as definitive biomarkers for particular ICNSs. Sixth, current techniques for circRNA detection, such as qPCR, RNA sequencing, or Northern blotting, may have limitations in sensitivity and specificity. Advanced techniques may be required to enhance detection capabilities, but these can be resource-intensive and not widely accessible. Addressing these challenges will be crucial for the successful translation of exosomal circRNAs into clinical practice for the diagnosis and management of ICNSs and other diseases.

In conclusion, our findings support for the emerging role of circRNAs in ICNSs and highlight the promise of brain-derived exosomal circRNAs as novel diagnostic markers for these challenging conditions. Further research in this field has the potential to revolutionize the diagnosis and management of ICNSs, ultimately improving patient outcomes and alleviating the burden of these devastating diseases.

## Figures and Tables

**Table 1:** Demographic characteristics of patients (patients) and control volunteers (controls).

Parameter	Control (*n* = 21)	Patients	
		Bacteria (*n* = 6)	Virus (*n* = 38)
Age	64.10 ± 11.28	57.67 ± 9.58	52.63 ± 19.04
Sex			
Male	10	3	24
Female	11	3	14
Blood			
White blood cell (count 10^9^/L)	6.89 ± 2.15	12.85 ± 5.98	8.05 ± 3.46
Neutrophil (count 10^9^/L)	4.37 ± 2.02	11.18 ± 6.15	6 ± 3.39
Lymphocyte count (10^9^/L)	1.97 ± 0.72	0.85 ± 0.36	1.67 ± 1.83
Hemoglobin (count 10^9^/L)	137.05 ± 15.69	126 ± 24.08	125.58 ± 21.32
Platelet count (10^9^/L)	187.38 ± 52.64	184.67 ± 72	183.71 ± 84.4
ALT (U/L)	22.74 ± 13.51	35.75 ± 23.97	36.67 ± 49.97
AST (U/L)	21.02 ± 9.56	21.25 ± 7.33	63.25 ± 119.69
T-BIL (μmol/L)	11.57 ± 10.64	17.85 ± 20.26	11.08 ± 5.61
Alb (g/L)	42.14 ± 3.16	38.25 ± 12.32	36.17 ± 4.88
Glu (mmol/L)	5.34 ± 1.34	8.05 ± 3.22	6.6 ± 4.25
Cr (μmol/L)	66.52 ± 15.6	71.33 ± 49.78	88.89 ± 112.77
Potassium K (mmol/L)	3.98 ± 0.41	3.88 ± 0.28	3.81 ± 0.45
Na (mmol/L)	142.06 ± 1.65	139.63 ± 2.88	152.55 ± 81.58
Ca (mmol/L)	2.31 ± 0.13	2.15 ± 0.09	2.49 ± 1.56
C-reaction protein (mg/L)	5.02 ± 5.79	110.72 ± 74.41	34.68 ± 44.29
Prothrombin time (s)	10.76 ± 0.58	12.87 ± 2	11.64 ± 1.49
INR	0.94 ± 0.05	1.13 ± 0.18	1.02 ± 0.14
TT (s)	18.49 ± 1.02	16.38 ± 1.23	17.49 ± 2.72
Fibrinogen (g/L)	2.49 ± 0.63	5.58 ± 1.65	4.85 ± 6.68
D-dimer (mg/L)	0.52 ± 0.61	1.97 ± 0.99	5.64 ± 13.17
CSF			
White blood cell (count 10^9^/L)	—	1585.67 ± 1554.35	34.88 ± 43.04
Neutrophil (count 10^9^/L)	—	1326.92 ± 1378.53	12.18 ± 25.46
Lymphocyte count (10^9^/L)	—	258.75 ± 251.06	20.82 ± 30.64
Glu (mM)	—	1.6 ± 0.94	4.01 ± 1.31
Cl (mM)	—	116.98 ± 13.96	125.68 ± 5.11
CSF totalprotein (mg/L)	—	3966.1 ± 2282.47	828.73 ± 593.4
IFN-*γ* (IU)	—	150.06 ± 138.65	18.98 ± 45.1
IgG	—	533.74 ± 292.02	97.05 ± 86.41
PCT (ng/mL)	—	1.78 ± 1.43	0.87 ± 2.48

Abbreviation: CSF, cerebrospinal fluid.

## Data Availability

The article/Supporting Information contains all the datasets produced for this investigation. The laboratory stores both raw and processed data, which can be obtained upon request.

## Supporting Information

Supporting Information Supporting information shows the tables displaying the gene names and expression abundances detected in three groups using high-throughput sequencing: healthy human controls, viral CNS infections, and bacterial CNS infections.
